# Distinctive emotional responses of clinicians to suicide-attempting patients - a comparative study

**DOI:** 10.1186/1471-244X-13-230

**Published:** 2013-09-22

**Authors:** Zimri S Yaseen, Jessica Briggs, Irina Kopeykina, Kali M Orchard, Jessica Silberlicht, Hetal Bhingradia, Igor I Galynker

**Affiliations:** 1 Department of Psychiatry, Beth Israel Medical Center, New York, NY 10003, USA

**Keywords:** Suicide, Suicidality, Suicide risk assessment, Patient death, Affect, Counter-transference questionnaire, Therapist response, Counter-transference, Counter-transference hate, Therapy

## Abstract

**Background:**

Clinician responses to patients have been recognized as an important factor in treatment outcome. Clinician responses to suicidal patients have received little attention in the literature however, and no quantitative studies have been published. Further, although patients with high versus low lethality suicidal behaviors have been speculated to represent two distinct populations, clinicians’ emotional responses to them have not been examined.

**Methods:**

Clinicians’ responses to their patients when last seeing them prior to patients’ suicide attempt or death were assessed retrospectively with the Therapist Response/Countertransference Questionnaire, administered anonymously via an Internet survey service. Scores on individual items and subscale scores were compared between groups, and linear discriminant analysis was applied to determine the combination of items that best discriminated between groups.

**Results:**

Clinicians reported on patients who completed suicide, made high-lethality attempts, low-lethality attempts, or died unexpected non-suicidal deaths in a total of 82 cases. We found that clinicians treating imminently suicidal patients had less positive feelings towards these patients than for non-suicidal patients, but had higher hopes for their treatment, while finding themselves notably more overwhelmed, distressed by, and to some degree avoidant of them. Further, we found that the specific paradoxical combination of hopefulness and distress/avoidance was a significant discriminator between suicidal patients and those who died unexpected non-suicidal deaths with 90% sensitivity and 56% specificity. In addition, we identified one questionnaire item that discriminated significantly between high- and low-lethality suicide patients.

**Conclusions:**

Clinicians’ emotional responses to patients at risk versus not at risk for imminent suicide attempt may be distinct in ways consistent with responses theorized by Maltsberger and Buie in 1974. Prospective replication is needed to confirm these results, however. Our findings demonstrate the feasibility of using quantitative self-report methodologies for investigation of the relationship between clinicians’ emotional responses to suicidal patients and suicide risk.

## Background

When treating patients at risk for suicide, clinicians often struggle to identify signs, symptoms, or precipitating events that might afford opportunities for them to intervene. Clinically, we remain largely unable to accurately distinguish between patients who will attempt or die by suicide and patients who will not [1,2]. Clinicians’ emotional responses to patients (broadly speaking, their countertransference) have long and increasingly been recognized as an important factor in treatment outcome [3,4], however they have received relatively little attention in the literature on suicidal patients. Rather, current research on acute suicide prediction has focused largely on warning signs that are patient-dependent, such as precipitating events [5-9], behavior changes [10,11], or intense affective states [12-19]. Yet, even though they are easily identified retrospectively, such findings may be difficult to utilize clinically; these markers may be masked and/or minimized by the patient, or misattributed/misinterpreted by the therapist [20-22], and in some cases overzealous efforts at intervention, such as those that prematurely push an unready patient towards independence, even appear to precipitate patient suicide [5].

A potential factor contributing to these difficulties, beyond the general difficulty of predicting human behavior, and external constraints of the current mental health care system (e.g. [23]), may lie in clinicians’ own emotional responses to the suicidal patient. While clinical judgment is ultimately a conscious process, the suicidal patient elicits powerful responses that may not become directly conscious [4,24]. Indeed, neuroimaging studies suggest activation of brain regions primarily involved in unconscious processing during emotional as compared to cognitive empathy tasks [25]. Without (and even with [26]) tremendous experience, unaided conscious integration of unconscious emotional responses is likely to fail. A systematic assessment of these responses, however, has the potential to ameliorate the inherent distortions of the clinician’s judgment without discarding the data inherent in his or her interpersonal experience with the patient.

Clinician-focused research supports distinctive patterns of reaction to various patient types [27-29], and there is a relatively large body of literature examining clinicians’ reactions following patient suicide (e.g., [22,30-32]) and unexpected death [33] which observe prominent reactions of grief and mourning on the one hand [30], and guilt and anger on the other [32,34]. Similarly, clinicians confronted with patients’ desire for death in studies of physician assisted suicide (also only qualitative), elicited anxious, helpless, and overwhelmed responses most prominently [35]. Clinicians’ emotional responses to suicidal patients have not been the subject of many research studies. Since Maltsberger and Buie’s seminal 1974 paper [24], which elaborated an array of emotional experiences and behaviors rooted in different defense responses to negative countertransference towards the suicidal patient, only a few empirical studies have been conducted. These retrospective clinical studies have focused almost exclusively on countertransference hate and/or negative countertransference in general, finding feelings of anxiety and hostility as those most prominently elicited by suicidal patients [34,36]. The studies share a common conclusion that emotional responses must be recognized and acknowledged, and present evidence that the management of the clinicians’ emotional response is correlated with therapeutic outcome [3,22,37]. Quantifying clinicians’ emotional responses may thus potentially enhance suicide risk assessment.

The present preliminary study, though conducted retrospectively, assessed clinicians’ reported emotional responses toward their patients in the encounter preceding their suicide attempt, completed suicide, or unexpected (non-suicide) death, with a focus on quantifying differences in the patterns of clinician response to patients with differing levels or types of suicidality. The goal was to identify potential significant differences in clinicians’ emotional responses to the patients that were at imminent risk for suicidal behavior, compared with those who were not. Ultimately, a thorough understanding of characteristic emotional responses to imminently suicidal patients might allow clinicians to better recognize those responses to their patients that might interfere with taking appropriate measures to prevent imminent suicidal actions, or that in themselves may serve as warning signs of imminent suicidality.

## Methods

An anonymous web-based survey (implemented through the surveymonkey.com website) was distributed to psychiatrists, psychologists, and social workers at the Beth Israel Medical Center in New York City via a department-wide email message requesting participation including the link to the anonymous survey. Participation occurred on a voluntary basis and participants had the ability to discontinue at any time. Participants were informed of the nature of the study in the email message inviting them to complete the survey. The study was approved by the Beth Israel Medical Center Institutional Review Board.

The survey consisted of the Therapist Response/Countertransference Questionnaire (CQ) – a 79-item self-report measure designed for clinicians which provides a validated instrument for assessing countertransference patterns in the psychotherapeutic setting [29], as well as questions regarding the demographic and clinical characteristics of the clinicians and the patients they reported on. The CQ has eight defined subscales (found to be independent of clinicians’ theoretical orientation): *overwhelmed-disorganized* (coefficient alpha = 0.90) “marked by items indicating a desire to avoid or flee the patient and strong negative feelings, including dread, repulsion, and resentment”, *helpless-inadequate* (coefficient alpha = 0.88), “describing feelings of inadequacy, incompetence, hopelessness, and anxiety”, *positive* (coefficient alpha = 0.86), “indicating the experience of a positive working alliance and close connection with the patient”, *special-over-involved* (coefficient alpha = 0.75), “describing a sense of the patient as special, relative to other patients, and … ‘soft signs’ of problems in maintaining boundaries”, *sexualized* (coefficient alpha = 0.77), “describing sexual feelings toward the patient or … sexual tension”, *disengaged* (coefficient alpha = 0.83), “describing feeling distracted, withdrawn, annoyed, or bored”, *parental-protective* (coefficient alpha = 0.80), “describing a wish to protect and nurture the patient in a parental way… beyond normal positive feelings”, and *criticized-mistreated* (coefficient alpha = 0.83), “describing feelings of being unappreciated, dismissed, or devalued” [29]. The CQ was used to assess countertransference in clinicians across four different patient categories: suicide completers, high-lethality suicide attempters (as indicated by clinical judgment and/or necessity for hospitalization), low-lethality suicide attempters (as indicated by clinical judgment), and patients who suffered sudden (unexpected) non-suicide death. The order of patient category presentation was randomized for each respondent. In each patient category the clinicians were prompted to fill out the questionnaire based on their experiences in regard to “the patient you remember best” in the last session preceding their suicide attempt or death. This prompt was chosen to elicit what, in the absence of prospective data, should be the most reliable. [38] If a clinician reported having treated a patient in more than one category, a separate CQ was filled out for each patient category individually. Clinicians were instructed to rate each item on the questionnaire as 1, 3, or 5, based on the extent to which it was true in their work with the patient in question; 1 = not true at all, 3 = somewhat true, and 5 = very true.

### Statistical analysis

Two group comparisons were performed: 1) any suicidal behavior versus unexpected deaths (SA vs. UD), and 2) high lethality and completed suicide attempts versus low lethality attempts (HL vs. LL). The first comparison was chosen to address the primary aim of the study, identification and quantification of any distinctive clinician response to patients presenting with imminent suicidality. The second comparison addresses a secondary question – ‘are there clinician responses distinctive of high lethality attempters versus low-lethality ones?’ in light of extensive literature suggesting clinical and biological differences between these groups [39]. High lethality attempts and completed suicides were combined as completed suicides result, by definition, from highly lethal attempts.

Unpaired two-tailed t-tests were used to compare group means on each of the eight defined CQ subscales. To assess clinician effects, these group comparisons were repeated restricted to the subsets of clinicians who reported on patients in both groups in each comparison. In the repeated analysis means were compared pair-wise by clinician using paired two-tailed t-tests. We report both conservative estimates of significance, using Bonferroni correction of criterion alphas, and uncorrected estimates, as the Bonferroni correction has been considered inappropriately stringent for medical research, biasing results towards type II error, and thus potentially obscuring useful findings [40].

To identify an effective subscale of items that might best discriminate between suicide attempters and non-attempters, and high versus low lethality attempters, stepwise linear discriminant analyses were used with a threshold p = 0.05 for variable inclusion and p = 0.10 for exclusion in the linear discriminant analysis. In the analysis, cases with no missing values for any scale item were used. Leave-one-out cross-validation of the discriminant function provided a measure of the difference between groups in their responses on the CQ that is robust to over-fitting of the data (and thus false positive findings). All of the above analyses were carried out using the SPSS software package.

In secondary analysis, to account for possible chronic differences in level of suicidal capacity [41] between patients who attempt suicide and those who do not, findings from the above analyses were stratified by presence or absence of a past history of suicide attempt, as a control for the effect of past history of suicidality.

Post hoc power analyses indicate that for the achieved sample sizes the study had 80-95% power to detect moderate to large effects (Cohen’s d = 0.63-0.84) for the “High versus Low Lethality (HL vs. LL)” comparisons of means, and large effects (Cohen’s d = 0.70-0.92) for the “Any Suicidality versus Unexpected Death (SA vs. UD)” comparisons of means at the p < 0.05 probability level. Given the necessarily high level of interpersonal variability in clinicians’ emotional reactions to patients, large effects are those of greatest clinical interest.

## Results

### Sample characteristics

Two hundred clinicians received the invitation email with the survey link. 83 (42%) clinicians began the web-based survey, and 40 (20% of those approached, 48.2% of those responding) provided CQ reports on a total of 82 patients. The clinicians assessed in the study showed a near equal split between males and females, and held a variety of higher-level degrees; though the most common by far was an MD (50%). A small majority of the clinicians assessed had been in practice for less than five years or more than twenty; those who had been practicing for between five and twenty years were slightly less likely to complete the questionnaire (See Table 1).

**Table 1:** Clinician demographics

		**Frequency (n)**	**Percent**
**Sex**	Male	19	47.5
	Female	21	52.5
**Degree**	MD	20	50.0
	PhD	7	17.5
	PsyD	2	5.0
	LCSW	5	12.5
	MSW	4	10.0
	Other	2	5.0
**Years in practice**	0-5	11	27.5
	5-10	7	17.5
	10-15	6	15.0
	15-20	4	10.0
	>20	12	30.0

Of 82 reports assessed, 16 were regarding patients that died unexpectedly, 26 were on patients who made low lethality suicide attempts, 28 were on patients that made high lethality suicide attempts, and 12 were on suicide completers. Patients who made suicide attempts (of any lethality level) were generally younger than those who completed suicide or died unexpectedly (independent groups *t*-test 2-tailed p = 0.01). Those who made low lethality suicide attempts were predominantly female (76%), while those in the other three groups were closer to evenly split along gender lines (chi square p = 0.04). In all four groups, the patients assessed were predominantly white (no significant differences using chi square statistics). Finally, the groups of patients who attempted suicide had more members with a history of past suicide attempt than members without such a history. Conversely, more of the patients who died unexpectedly did not have a history of suicide attempt, and the patients who completed suicide were evenly split. These group differences were not statistically significant (using chi square statistics) however (See Table 2).

**Table 2:** Patient demographics

		**Unexpected death**		**Low lethality SA**		**High lethality SA**		**Completed suicide**	
		**n**	**Valid %**	**n**	**Valid %**	**n**	**Valid %**	**n**	**Valid %**
**Sex**	Male	6	40.0	6	24.0	14	51.9	5	45.5
	Female	9	60.0	19	76.0	13	48.1	6	54.5
	Not reported	1	NA	1	NA	1	NA	1	NA
**Race**	Asian	0	0.0	0	0.0	2	7.4	1	9.0
	Am. Indian/Pac. Islander	1	6.7	1	4.0	0	0.0	0	0.0
	Black	2	13.3	2	8.0	5	18.5	1	9.0
	White	8	53.3	20	80.0	18	66.7	9	81.8
	Other	4	26.7	2	8.0	1	3.7	0	0.0
	Not disclosed	0	0.0	0	0.0	1	3.7	0	0.0
	Not reported	1	NA	1	NA	1	NA	1	NA
**Primary Dx**	Schizophrenia/Schizoaffective	4	26.7	2	8.0	5	18.5	3	27.3
	Bipolar I or II	2	13.3	7	28.0	8	29.6	2	18.2
	Unipolar depression	4	26.7	9	36.0	7	25.9	4	36.4
	Personality disorder	2	13.3	6	24.0	5	18.5	2	18.2
	Anxiety disorder	3	20.0	1	4.0	0	0.0	0	0.0
	Substance abuse	0	0.0	0	0.0	1	3.7	0	0.0
	Unknown	0	0.0	0	0.0	0	0.0	0	0.0
	Other	0	0.0	0	0.0	1	3.7	0	0.0
	Not reported	1	NA	1	NA	1	NA	1	NA
**Past suicide attempts**	Yes	5	35.7	15	60.0	15	55.6	5	45.5
	No	9	64.3	10	40.0	12	44.4	6	54.5
	Not reported	2	NA	1	NA	1	NA	1	NA
**Length of therapy**	Mode	1-5 years		1-5 years		5 years		1 year	
	Approx. median	1 year		6 months – 1 year		1-5 years		1 year	
**Mean age**		49.7 years		37.2 years		41.3 years		49.1 years	

### Group contrasts -- SA vs. UD

For the SA vs. UD group comparison of the mean scores on each of the eight defined subscales of the Therapist Response/Countertransference Questionnaire, one subscale differed significantly and one approached significance. Mean scores were 5.95 points (p = 0.005, criterion alpha corrected for 8 comparisons = 0.0063) higher on the “Overwhelmed/Disorganized” subscale, and 2.54 points (p = 0.054) higher on the “Hostile/Mistreated” subscales for the SA group. No differences approached significance on the other subscales. Thirteen clinicians reported on both SA and UD patients. T-test comparison of SA versus UD means for each subscale paired by clinician replicated the overall results with mean difference 7.00 points (p = 0.023) on the “Overwhelmed/Disorganized” subscale and 2.77 points (p = 0.056) on the “Hostile/Mistreated” subscale, and no differences approaching significance (p < 0.1) on the other subscales.

Eight individual questionnaire items differed significantly (using uncorrected criterion alpha = 0.05) between the SA and UD groups. The strongest effects were found for positive (in the sense of affiliative or approach-promoting) therapist response items, which, though generally rated highly, had significantly lower ratings for suicidal patients. Likewise, negative (in the sense of aggression or withdrawal-promoting) therapist response items were rated more highly for suicidal patients than for non-attempters, though in both cases the means fell between “somewhat” and “not at all”. For suicidal patients, mean score on the item “I liked him/her very much” was higher than that for any other item differing significantly from non-attempters. Self-report of sexualized therapist response was very low for all groups of patients; it was lower for patients that attempted or completed suicide than for non-attempters, however this difference may be driven by outliers in light of the small variances in the samples. No items differed with p-value less than criterion alpha corrected for 79 comparisons (alpha = 0.0006) (See Table 3).

**Table 3:** CQ items differing most strongly for SA vs. UD comparison

**CQ item**	**SA group mean**	**UD group mean**	**F-value**	**P-value**
23. S/he made me feel good about myself.	1.89	3.00	9.09	.004
65. I liked him/her very much.	2.48	3.83	7.70	.007
5. I wished I had never taken him/her on as a patient.	2.33	1.17	6.50	.013
59. I felt like my hands were tied or that I was put in an impossible bind.	2.33	1.17	5.79	.019
17. I felt sexually attracted to him/her.	1.04	1.33	5.21	.026
6. I felt dismissed or devalued.	2.19	1.17	4.80	.032
70. I returned his/her phone calls less promptly than I did with my other patients.	1.74	1.00	4.64	.035
24. I felt guilty about my feelings toward him/her.	1.93	1.17	4.05	.048

When analysis was stratified by history of past suicide attempts we found that of these eight items, among patients with no past history of suicide attempt, the differences remained statistically significant for all but two items: “24. I felt guilty about my feelings toward him/her” and item “5. I returned his/her phone calls less promptly than I did with my other patients”. Among patients with a past history of suicide attempt, no difference in means was statistically significant. This analysis was limited by the small number (4 patients) of patients in the UD group with a history of past suicide attempt(s).

Stepwise linear discriminant analysis for the SA vs. UD group comparison produced a discriminant function derived from scores on five items: “1. I am very hopeful about the gains s/he is making or will likely make in treatment”, canonical discriminant function coefficient 0.498, SA > UD, “23. S/he makes me feel good about myself”, coefficient −0.939, SA < UD, “52. I feel hopeless working with him/her”, coefficient −0.672, SA < UD, “70. I return his/her phone calls less promptly than I do with my other patients”, coefficient 0.629, SA > UD, and “79. I talk about him/her with my spouse or significant other more than my other patients”, coefficient 0.563, SA > UD. The discriminant function thus describes a combination of greater avowed hopefulness combined with more negative feelings about self, avoidance of the patient, and comfort seeking behavior by the clinician in treating suicidal patients. This discriminant function classified SA vs. UD patients with an 87.8% cross-validated correct classification rate (Chi-squared = 23.58, p < 0.0001), with 90% sensitivity and 56% specificity for suicidal patients (See Table 4).

**Table 4:** Discriminant analysis classification table: UD vs. SA

		**Predicted group membership**	
**Actual group membership (below):**		**Unexpected death**	**Any suicidality**
**Original classification**	Unexpected death	10	6
	Any suicidality	3	63
**Cross-validated**^**a**^	Unexpected death	9	7
	Any suicidality	3	63

^a^In cross validation, each case is classified by the functions derived from all cases other than that case.

T-test comparison of SA versus UD means for discriminant function score, paired by clinician, replicated the overall results with a highly significant mean difference 1.77 points (p = 0.0003).

Further, when this analysis was stratified by history of SA, the discrimination was significant both when history of SA was present and when it was not. When history of SA was present, the cross-validated correct classification rate was 97.1% (Chi-squared = 21.71, p = 0.001), with sensitivity of 100% and specificity of 66.7% for suicidal patients. When history of SA was not present, the cross-validated correct classification rate was 78.8% (Chi-squared = 12.76, p = 0.026), with sensitivity of 84.0% and specificity of 62.5% for suicidal patients.

### Group contrasts -- HL vs. LL

In the HL vs. LL group comparison of the mean scores on each of the eight defined subscales of the Therapist Response/Countertransference Questionnaire, no significant differences were found. The greatest difference in means was found for the “Positive/Satisfying” response scale, which was 2.6 points higher for the HL group (p = 0.18).

In clinician-wise paired t-tests on matched cases from 17 clinicians reporting on both HL and LL patients no significant differences were found. The greatest mean difference was found for the “Parental/Protective” subscale which averaged 3.1 points higher for the HL group (p = 0.07).

Comparison of the mean scores on each item of the Therapist Response/Countertransference Questionnaire found one item – “49. I felt sad in sessions with him/her” that differed with p < 0.05 between HL and LL groups (means 2.69 and 1.82, respectively; p = .024). In clinician-wise paired *t*-test means for this item were 2.82 and 1.94 respectively, p = 0.039. When analysis was stratified by history of past suicide attempts, we found that the mean score on item “49”differed significantly between HL and LL groups only for patients who had a past history of SA (means 3.00 and 1.43, respectively; p = .001). No items differed significantly after Bonferroni correction for 79 comparisons.

Four CQ items describing depression, guilt and helplessness had strong correlations (r > 0.5) with this item: “18. I feel depressed in sessions with him/her” (r = 0.665), “28. I feel guilty when s/he is distressed or deteriorates, as if I must be somehow responsible” (r = 0.575), “24. I feel guilty about my feelings toward him/her” (r = 0.528), and “26. I feel overwhelmed by his/her strong emotions” (r = 0.508). Group means for these items did not differ between groups at the 0.05 significance level, however, and they were thus excluded from the discriminant analysis.

Stepwise linear discriminant analysis for the HL vs. LL group comparison thus produced a discriminant function derived from scores on the single item – “49. I felt sad in sessions with him/her” – that classified high lethality suicidal behavior (high lethality attempts and completed suicides) versus low lethality suicide attempts with modest but statistically significant power. The cross-validated correct classification rate was 66.7% (Chi-squared = 5.19, p = 0.023), with sensitivity of 70% and specificity of 61.5% for high lethality and completed suicide. Application of the discriminant function to patients with unexpected non-suicide death resulted in random assignment of predicted group membership (50% predicted to each group) (See Table 5).

**Table 5:** Discriminant analysis classification table: HL vs. LL

		**Predicted group membership:**	
**Actual group membership (below):**		**Low lethality SA**	**High lethality or completed SA**
**Original classification**	Low lethality SA	16	10
	High lethality or completed SA	12	28
**Cross-validated**^**a**^	Low lethality SA	16	10
	High lethality or completed SA	12	28
**Classification of excluded cases**	Unexpected death	8	8

^a^Cross validation is done only for those cases in the analysis. In cross validation, each case is classified by the functions derived from all cases other than that case.

When this analysis was stratified by history of SA, the discrimination was significant only when a past history of SA was present. Among patients with a past history of suicide attempts, the cross-validated correct classification rate was improved to 76.5% (Chi-squared = 10.86, p = 0.001), with sensitivity of 75% and specificity of 78.6% for high lethality and completed suicide.

## Discussion

To the best of our knowledge, this is the first published study to provide a quantitative comparison of clinician responses to acutely suicidal patients versus non-attempters and to patients who made high lethality versus low-lethality suicide attempts.

Such investigation is important, as problems in the management of countertransference (or emotional reactions in general) to patients may hamper treatment efficacy and even contribute to patient suicide in a small but significant proportion of cases [5,36]. To date though, the literature has focused almost entirely on the development of qualitative treatments of the subject. A thorough literature search using the PsycINFO database resulted in our conclusion that there are no analogous studies in the literature. (Searches conducted using varied combinations of terms including “countertransference”, “suicide”, “therapist response”, “clinician response”, “predict”, “prevention”, “comparison”, and “quantitative” identified no peer-reviewed publications reporting on quantitative comparisons of clinician responses to suicidal versus non-suicidal patients or of patients with differing levels of suicidality). The only quantitative comparative work we have been able to find on the subject has been a small series of unpublished dissertations, which found no significant differences in negative therapist responses to suicidal versus “difficult” non-suicidal patients [42]. While rich qualitative data are an essential starting point, this preliminary study aimed to pilot a much-needed quantitative and comparative approach using a validated instrument and easily replicable quantitative methodology.

This study found that clinicians treating imminently suicidal patients recalled, on average, moderately positive feelings towards these patients (though less so than for non-attempters), with higher hopes for treatment, while finding themselves more overwhelmed, distressed by, and, at low levels, avoidant of them. Further, we found that the specific paradoxical combination of hopefulness and distress/avoidance was a significant discriminator between suicidal patients and those who had unexpected non-suicide deaths, and cross-validated classification by discriminant analysis remained statistically significant both when a past history of suicide attempt was present and when it was not. This finding of ‘paradoxical response’ is consistent with the higher scores observed on the “overwhelmed-disorganized” subscale of the CQ in clinician recollections of their encounters with suicide attempters.

In our second comparison, we found no clear evidence of differences between clinicians’ responses in encounters with patients preceding either completed suicides or highly lethal suicide attempts and their responses in encounters preceding low lethality suicide attempts. Despite a trend towards a slightly more positive emotional responses overall, clinicians also recalled experiencing more sadness in encounters with patients preceding either successful or highly lethal suicide attempts than in encounters preceding low lethality suicide attempts. This difference in recalled sadness was found to be a modest discriminator between patients that went on to exhibit high and low lethality suicidal behavior. It is worth noting, however, that this difference appears attributable specifically to recalled responses to patients with a history of previous attempts; among these patients sadness in session was a significant discriminator of attempt lethality while among first-time attempters clinicians’ experience of sadness in the session prior to suicide attempt did not differentiate between lethality levels. This finding has not been supported or opposed in the literature, as the difference in emotional response to high and low lethality suicide attempters has not previously been explored. Further, interpretation is limited by significant risk of type-1 error given the small n’s and multiple comparisons involved.

Thus our findings, while grossly consistent with the qualitative literature findings of negative responses to suicidal patients [34,36], differed in the important respect that the levels of recalled negative reactions to patients prior to their suicide attempts were, on average, fairly low, and even when significant, the magnitude of the differences in positive and negative responses elicited by suicidal versus non-suicidal patients was small. Maltsberger and Buie [24] were the first to describe in detail the negative countertransference (“countertransference hate”) that clinicians may experience in response to suicidal patients, and provided a theoretical framework which might account for our quantitative findings. First, as noted, we found that clinicians recalled fairly low levels of negative feelings towards their suicidal patients, though positive response was attenuated compared to non-attempters. This finding may be consistent with their predictions of repression of “countertransference hate”. On the other hand, our findings of distress and self-directed negative feelings combined with paradoxical hopefulness may be consistent with their predictions of turning of the countertransferential hate against the self and of reaction-formation against it, respectively. Indeed, our findings seem to suggest that the defense mechanisms described by Maltsberger and Buie may operate in concert.

Our findings point to the potential clinical utility of self-assessment of emotional response in the treatment of suicidal patients. This is a matter of some importance as both Modestin [36] and Marcinko et al., [22] have used observational evidence to support the theory that emotional responses to suicidal patients that are not properly managed can have harmful consequences. The latter group concluded that negative emotional response probably contributes to or correlates with negative patient outcomes [22], while Modestin, further indicates how the failure to control these reactions (hostility, hate, and aggressiveness in particular) may in some cases help push patients to suicide [36].

We should note, however, that while both emotional responses and judgments of suicide risk reside in the clinician, they are not the same. Indeed clinical judgment has been found to be a poor predictor of critical patient behavior such as suicide [1] and violence [43]. While clinical judgment is ultimately a conscious process, emotional responses may not become directly conscious [4,24]. Thus systematic assessment of these responses, even in using self-report measures may reveal patterns generated by the clinician’s unconscious processes such as the “paradoxical hopefulness” identified using discriminant analysis. Quantitative self-report assessment may thus reveal data inherent in the clinician’s interpersonal experience with the patient that could potentially augment suicide-risk assessment.

### Limitations

The results of this preliminary study must be considered in light of several important limitations. Most prominently the study is subject to several kinds of recall bias.

First, many clinicians that have experienced a patient’s death by suicide report severe distress [32] and/or feelings of grief and self-doubt [31] stemming from treatment decisions that seem, in retrospect, to have been based on inaccurate assessments of the patient’s acute risk. Differences between such responses to patients’ suicide deaths, attempts of different severity, and unexpected non-suicide deaths have not been studied and are poorly understood [33]. It is possible that the differences in recalled reaction to patients in the encounters preceding such events are attributable to their recollection being colored differently by those very events. Furthermore, individual items in the CQ might be differently subject to such effects thereby increasing or decreasing their apparent discriminatory power in our results.

Second, clinicians’ recollection of their responses to their patients in the encounters immediately preceding such events are almost certainly significantly combined with the rest of their preceding experience with those patients. Thus our findings cannot be interpreted as necessarily indicative of a “pre-suicidal” countertransference or emotional response.

Third, we are unable to control for the possible effects of clinicians’ reporting on their best-remembered patient of each type. Additionally, we were not able to control for the effects on recall of time elapsed since the events.

Fourth, as we were unable to obtain responses on each category of patient from most clinicians, it is possible that clinicians responding on suicidal patients we more likely to treat suicidal patients and thus represented a distinct group from those responding regarding non-suicidal patients only. Thus it is conceivable that differences in response are attributable to clinician differences rather than patient ones. However, the consistency between aggregate group findings and the within-clinician findings, for those clinicians who reported on patients belonging to different comparison groups, makes such an interpretation less likely.

Further, because the survey was distributed only within one institution, and was completed voluntarily, we cannot say that it accurately represents all clinicians who have experienced a patients’ completed suicide, attempt, or unexpected death.

Finally, limitations of sample size did not allow for reliable analysis of potential mediators and moderators of differences in therapist responses to patients of different types. Nonetheless it should be noted that no statistically significant differences in the rates of any diagnostic or demographic characteristics were observed between groups.

In sum, our findings must be viewed as preliminary results that justify further research. In order to more definitively verify our conclusions, the study will need to be repeated with a wider, larger sample. Additionally, prospective replication is necessary to confirm the findings.

## Conclusions

We find preliminary quantitative evidence consistent with Maltsberger and Buie’s theory of countertransference hate in the treatment of suicidal patients. Though our study does not speak to the ability of the differences in response to influence or predict a patient’s outcome, it is the first to quantify the differences in clinicians’ emotional responses to suicidal patients versus non-attempter patients. Our findings thus provide a starting point for further research that may change the way that clinicians assess their suicidal patients’ acute risk, and may justify further research on the use of the CQ or other conceptually similar scales as predictors of suicide risk.

## Competing interests

The authors declare that they have no competing interests.

## Authors’ contributions

IK, KMO, JS, HB & IIG participated in the design of the experiment. IK and IIG collected the data. ZSY designed and performed the data analysis. JB and IK prepared the data. ZSY, JB, and IIG participated in the writing of the manuscript. All authors read and approved the final manuscript.

## Authors’ information

Jessica Briggs is co-first author.

## Pre-publication history

The pre-publication history for this paper can be accessed here:

http://www.biomedcentral.com/1471-244X/13/230/prepub
